# Prognostic assessment of comatose patients after cardiac arrest: construction of a simplified monitoring EEG risk score across different time windows

**DOI:** 10.3389/fneur.2026.1812084

**Published:** 2026-08-25

**Authors:** Yan Wang, Yuanhui Sheng, Feng Li

**Affiliations:** 1 Department of Neurology & Nursing Department, The First Affiliated Hospital of Chongqing Medical University, Chongqing, China; 2 Department of Critical Care Medicine & Department of Emergency, The First Affiliated Hospital of Chongqing Medical University, Chongqing, China

**Keywords:** cardiac arrest, coma, electroencephalogram, prognosis, risk assessment

## Abstract

**Objectives:**

To develop a Simplified EEG Risk Score (SERS) for accurate and time-robust prognostic assessment in comatose patients after cardiac arrest.

**Methods:**

This retrospective cohort study enrolled comatose patients after cardiac arrest admitted to the ICU of the First Affiliated Hospital of Chongqing Medical University between January 2020 and December 2024. Univariate logistic regression was used to derive β-coefficients for EEG features, including background amplitude, dominant frequency, continuity, reactivity, and sleep elements, which were incorporated into the SERS. Multivariate logistic regression identified independent predictors of poor neurological outcome. Prognostic performance was evaluated at Day 1, Days 2–5, and >5 days after cardiac arrest using receiver operating characteristic curves, with assessment of the area under the curve (AUC), accuracy, sensitivity, and specificity.

**Results:**

A total of 251 comatose patients after cardiac arrest were included; 45 (18%) had good outcomes and 206 (82%) had poor outcomes. Abnormal background amplitude, slow dominant frequency (δ/θ), discontinuous background, absent reactivity, and absent sleep waveforms were all significantly associated with poor prognosis (*P* < 0.001). The AUCs of SERS were 0.912, 0.893, and 0.881 at Day 1, Days 2–5, and >5 days, respectively, outperforming individual EEG features, commonly used EEG scores, and traditional clinical predictors. Risk stratification demonstrated clear separation of prognosis across low-, medium-, and high-risk groups at all time windows.

**Conclusions:**

SERS shows high predictive accuracy and temporal stability, enabling reliable risk stratification for poor neurological outcomes in comatose patients after cardiac arrest, supporting its potential clinical utility.

## 1 Introduction

Cardiac arrest (CA) is a major global health burden, with an annual incidence of 50–110 cases per 100,000 population (1, 2). Despite continuous advances in critical-care techniques, 40%−66 % of patients survive with severe neurological sequelae attributable to post-anoxic brain injury (3, 4), imposing a heavy burden on patients' families and healthcare resources (5, 6). Therefore, early and accurate prognostication is essential for formulating scientific clinical treatment plans and end-of-life decisions.

Electroencephalogram (EEG) captures real-time dynamic changes in cerebral function and is exquisitely sensitive to ischemic–hypoxic insults, enabling early identification of the severity of brain damage and potential recovery trajectories. Current evidence indicates that specific EEG features are strongly associated with neurological outcome: continuous background activity, preserved EEG reactivity, and preserved sleep-related waveforms usually herald a favorable prognosis (7, 8), whereas discontinuous background activity and loss of reactivity are closely linked to poor outcome (9, 10). However, these high-value predictive features are present only in selected subgroups. Approximately 40 % of comatose post-CA patients fall into a “prognostic gray zone”—their EEGs neither meet criteria for irreversible brain injury nor provide definitive evidence of recovery, leaving them in an indeterminate prognostic state (11, 12).

Moreover, the pathophysiological evolution of post-CA cerebral injury is highly dynamic (13, 14), and clinical interventions such as targeted temperature management (TTM) and the use of sedative–analgesic agents can further alter EEG features by modulating cerebral metabolism and neural-network activity (15, 16). These factors collectively introduce marked spatiotemporal heterogeneity into post-CA brain-function assessment, making single-time-point EEG patterns insufficient to fully capture the evolving injury.

This study therefore aims to integrate key EEG characteristics—including background activity, reactivity, epileptiform discharges, and sleep-related waveforms—across distinct time windows (Day 1, Days 2–5, and > Day 5) in comatose post-CA patients, and to construct a multi-feature Simplified EEG Risk Score (SERS) for clinical decision support.

## 2 Methods

### 2.1 Study population

This study included 251 comatose patients after CA admitted to the intensive care unit (ICU) of the First Affiliated Hospital of Chongqing Medical University from January 2020 to December 2024. The inclusion criteria were as follows: (1) age ≥18 years; (2) Glasgow Coma Scale (GCS) score ≤ 8 at admission. The exclusion criteria were as follows: (1) missing clinical data; (2) poor quality of EEG data, making EEG analysis and processing impossible; (3) continuous administration of sedative or anesthetic medications during EEG acquisition. This study was approved by the Medical Ethics Committee of the First Affiliated Hospital of Chongqing Medical University (approval number: 2024-179-01). Since a retrospective design was applied and clinical data were stored and evaluated anonymously, no additional informed consent was required.

### 2.2 Treatment protocol

All patients received standardized treatment according to the Chinese expert consensus on post-CA treatment (17). Hemodynamic stability was maintained after resuscitation, optimizing vital parameters and addressing the causes and triggers of CA. This included: airway management, with tracheal intubation for patients who had not regained spontaneous breathing or were in a coma to maintain airway patency and ventilation oxygenation, with mechanical ventilation if necessary to maintain oxygen partial pressure and carbon dioxide partial pressure; circulatory support, monitoring blood pressure, heart rate, and rhythm, with a systolic blood pressure not lower than 90 mmHg and a mean arterial pressure not lower than 65 mmHg, and volume resuscitation for patients in shock, actively managing arrhythmias; target temperature management, with core temperature controlled at 34–36 °C for at least 24 h after successful resuscitation in comatose patients, unless contraindicated (i.e., active bleeding or hemodynamic compromise during cooling); during temperature management, propofol or midazolam was used for sedation, and morphine, fentanyl, or remifentanil was used for analgesia, with gradual tapering when the temperature returned to normal; seizures and status epilepticus were allowed to be treated and managed, with diagnosis and treatment procedures following the guidelines of the American Epilepsy Society and the Neurocritical Care Society (18, 19). If patients had irreversible neurological injury or other severe complications with an extremely poor prognosis, withdrawal of life-sustaining treatment could be considered after multidisciplinary team assessment and full communication with the patient's family.

### 2.3 Clinical data collection

#### 2.3.1 Demographic characteristics

Age, gender.

#### 2.3.2 Resuscitation details

Location of CA occurrence, cause of CA, time to return of spontaneous circulation, initial rhythm.

#### 2.3.3 General clinical indicators

within 72 h after CA, including Glasgow Coma Scale (GCS) score, Charlson Comorbidity Index (CCI), Acute Physiology and Chronic Health Evaluation II (APACHE II) score, Sequential Organ Failure Assessment (SOFA) score, brainstem reflexes (pupillary light, corneal reflex), cranial CT or MRI, and seizure events and status epilepticus occurring during the hospital stay.

#### 2.3.4 Clinical biochemical indicators

the highest or lowest values obtained within 72 h after CA, including blood gas analysis (PH, PO2, PCO2, Na, K, Ca, Lac, SO2), white blood cell count, albumin, creatinine, prothrombin time, neuron-specific enolase (NSE), and S-100β protein.

#### 2.3.5 Common EEG scores

Synek grading, Young grading.

#### 2.3.6 EEG features

Background amplitude, background dominant frequency, background continuity, background symmetry; reactivity, SIRPID; epileptiform discharges; sleep waveforms.

#### 2.3.7 Outcome indicator

Glasgow-Pittsburgh Cerebral Performance Category (CPC) score.

### 2.4 EEG recording and definitions

In this study, EEG data were collected from scalp EEG signals, with silver/silver chloride electrodes placed according to the international 10/20 system. When the notch filter was set at 50 Hz, the pass filter range was 0.1–70 Hz, the EEG interpretation sensitivity was 10 μV/mm, the paper speed was 3 cm/s, and 19 digital channels were obtained, with electrocardiogram leads added. The electroencephalographs used for recording were the Nicolet One from Natus and the Easy III from Cadwell. Each EEG recording lasted for at least 2 h and was discontinued when patients regained consciousness or died. This study utilized prolonged EEG recordings with a minimum duration of 2 h rather than continuous EEG (cEEG) monitoring. To minimize the influence of medications on EEG interpretation, recordings were performed after discontinuation of sedative and anesthetic agents whenever clinically feasible. Due to the retrospective study design, the timing and frequency of EEG examinations were determined by treating physicians according to clinical requirements and patient conditions rather than a predefined study protocol. Consequently, the number and timing of EEG recordings varied among patients. For analysis, EEG recordings were categorized into predefined post-cardiac arrest time windows (Day 1, Days 2–5, and >Day 5), and recordings obtained within each time window were evaluated.

The definition and classification of EEG referred to the standardized terminology for critical care EEG established by the American Clinical Neurophysiology Society (ACNS) in 2021 (20) (see Supplemental Table 1). The background amplitude was divided into two groups: normal and abnormal, while the background continuity was divided into two groups: continuous and discontinuous. The background EEG activity was classified into two groups: a fast frequency band (α/β, defined as α 8–13 Hz or β >13 Hz) and a slow frequency band (θ/δ or absence of activity, defined as θ 4– < 8 Hz, δ 0.5– < 4 Hz, or no discernible EEG activity). The background symmetry was divided into three groups: symmetric, mildly asymmetric (amplitude asymmetry, bilateral difference < 50%, or frequency asymmetry, two-sided difference 0.5–1 Hz), and markedly asymmetric (bilateral amplitude difference ≥50% or frequency difference > 1 Hz). In this study, EEG reactivity was defined as changes in EEG caused by stimulation. When appropriate external stimuli were given to the patient, including noxious (pinching the nipple or pressing the supraorbital notch), visual (opening the eyes under light), and auditory (shouting the patient's name) (21, 22), a change in amplitude or frequency on the EEG indicated the presence of EEG reactivity; otherwise, EEG reactivity was absent. Electromyographic activity or blink artifacts were not considered reactivity. Among them, stimulus-induced rhythmic, periodic, or ictal discharges (SIRPIDs) refer to rhythmic, periodic, or ictal abnormal discharges that are induced mainly by alertness stimuli, such as auditory stimulation, chest compression, physical examination, sputum suction, turning, and other nursing work (23, 24). Vertex sharp waves, spindles, and K-complexes were defined as sleep-related waveforms because these waveforms are easily identifiable and serve as stage 1 and 2 sleep markers. If all three of the above waveforms disappeared, they were defined as the absence of sleep-related waveforms. If one or more waveforms were recorded during monitoring, this was defined as the presence of sleep-related waveforms. All categorical EEG variables were obtained through simultaneous review by two doctors. In most cases, the values of categorical EEG variables were the same or the average of the two interpretations. When subjective interpretations differed significantly, a third interpreter adjudicated, and the resulting value of the disputed variable was the average of the two closest interpretations.

### 2.5 Prognostic assessment

Patients were divided into good prognosis group (CPC 1-2) and poor prognosis group (CPC 3-5) based on the CPC score at discharge.

### 2.6 Statistical methods

Normally distributed measurement data were described using mean ± standard deviation, with independent sample *t*-tests for comparisons between the poor prognosis group and the good prognosis group. Skewed distribution measurement data were described using median and interquartile range, with comparisons between groups using the Mann-Whitney *U*-test. Categorical data were described using counts and rates, with comparisons between groups using chi-square tests or Fisher's exact tests.

To enhance the clinical applicability of the model and simplify complex model parameters, a SERS was established based on the regression coefficients (β values) of EEG features from univariate logistic regression models for poor prognosis. Scores were assigned as the closest integer or half-integer to the β values, and the total score was obtained by summing the scores of all individual EEG features. Patients were then categorized into low-risk, medium-risk, and high-risk groups based on the relationship between the total score and the risk of poor prognosis.

Multivariate logistic regression models were used to explore factors influencing poor prognosis and to construct predictive models. Leave-one-out cross-validation was performed for internal validation of the models. Due to differences in the number of subjects in each time window, three separate predictive models were constructed for the time windows of Day 1, Day 2–5, and >Day 5 respectively. The predictive performance of the models was evaluated using the area under the ROC curve (AUC), accuracy, sensitivity, and specificity. The integrated discrimination improvement (IDI) and net reclassification improvement (NRI) indicators were used to compare the predictive performance of different models.

All analyses were completed using SAS 9.4 software (Copyright ©2016 SAS Institute Inc. Cary, NC, USA). A *P*-value of < 0.05 was considered statistically significant.

## 3 Results

### 3.1 Comparison of clinical characteristics of patients

**Figure 1:**
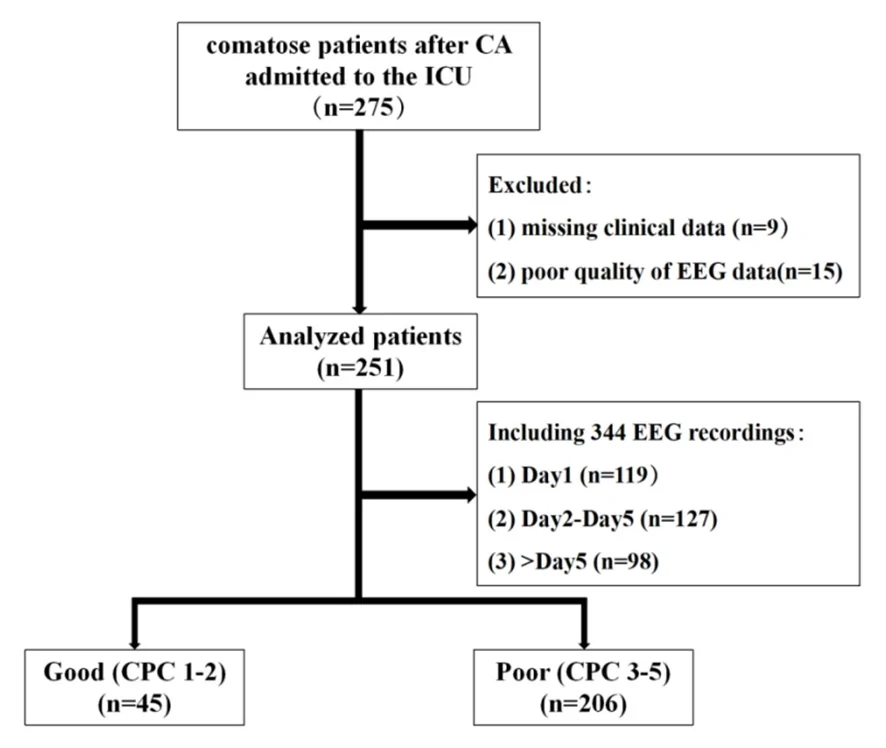
Recruitment flowchart.

A total of 251 comatose patients after CA were included in this study, with 45 (18%) having a good prognosis and 206 (82%) having a poor prognosis (see Figure 1). A total of 344 EEG recordings were collected, including 119 recordings within the first day after CA, 127 recordings during days 2–5, and 98 recordings beyond day 5. Table 1 shows the relationship between prognosis and patient clinical characteristics. There were statistically significant differences between the two groups in age, initial rhythm, CCI score, GCS score, APACHE II score, pupillary light reflex, corneal reflex, head CT/MRI results, creatinine, prothrombin time, NSE, and S-100β (all *P* < 0.05).

**Table 1:** Relationship between prognosis and clinical characteristics of comatose patients after cardiac arrest.

Variables	Total	Good prognosis (*n* = 45)	Poor prognosis (*n* = 206)	*t/Z/χ2*	*P*
Demographic characteristics					
Age, years	59.04 ± 18.68	52.22 ± 18.73	60.53 ± 18.37	−2.740	0.007
Gender					
Male	153 (60.96)	28 (62.22)	125 (60.68)	0.037	0.848
Female	98 (39.04)	17 (37.78)	81 (39.32)		
Resuscitation details					
Location of CA occurrence					
In-hospital	158 (62.95)	30 (66.67)	128 (62.14)	0.325	0.569
Out-of-hospital	93 (37.05)	15 (33.33)	78 (37.86)		
Cause of CA					
Non-cardiac	133 (52.99)	21 (46.67)	112 (54.37)	0.880	0.348
Cardiac	118 (47.01)	24 (53.33)	94 (45.63)		
Initial rhythm					
Non-ventricular fibrillation	199 (79.28)	28 (62.22)	171 (83.01)	9.716	< 0.001
Ventricular fibrillation	52 (20.72)	17 (37.78)	35 (16.99)		
Time to return of spontaneous circulation, min	13 (6, 31)	10 (3, 30)	13 (7, 31)	−1.460	0.144
Clinical general indicators					
CCI score	3.71 ± 2.85	2.71 ± 2.54	3.92 ± 2.88	−2.610	0.010
GCS score	5 (3, 8)	8 (5, 8)	5 (3, 7)	4.697	< 0.001
qSOFA score	2 (1, 2)	2 (1, 2)	2 (1, 2)	0.032	0.974
APACHE II score	27.73 ± 9.21	21.42 ± 7.90	29.10 ± 8.91	−5.341	< 0.001
Pupillary light reflex					
Absent	90 (35.86)	2 (4.44)	88 (42.72)	23.523	< 0.001
Present	161 (64.14)	43 (95.56)	118 (57.28)		
Corneal reflex					
Absent	146 (58.17)	8 (17.78)	138 (66.99)	36.759	< 0.001
Present	105 (41.83)	37 (82.22)	68 (33.01)		
Cranial CT/MRI results					
Normal	65 (26.75)	22 (50.00)	43 (21.61)	14.824	< 0.001
Abnormal	178 (73.25)	22 (50.00)	156 (78.39)		
Seizure events					
No	203 (80.88)	40 (88.89)	163 (79.13)	2.276	0.131
Yes	48 (19.12)	5 (11.11)	43 (20.87)		
Status epilepticus					
No	239 (95.22)	44 (97.78)	195 (94.66)	0.252	0.615
Yes	12 (4.78)	1 (2.22)	11 (5.34)		
Clinical biochemical indicators					
PH	7.34 ± 0.17	7.37 ± 0.12	7.33 ± 0.18	1.762	0.081
PCO_2_/mmHg	38 (33, 49)	37 (33, 42)	38 (33, 51)	−1.161	0.246
PO_2_/mmHg	128 (80, 199)	105 (77, 183)	133.50 (83, 200)	−1.012	0.312
Na/mmol/L	140.28 ± 11.52	139.91 ± 5.40	140.36 ± 12.47	−0.377	0.707
K/mmol/L	3.90 (3.40, 4.40)	3.70 (3.30, 4.20)	3.90 (3.50, 4.40)	−1.446	0.148
Ca/mmol/L	1.16 (1.05, 1.88)	1.12 (1.03, 1.78)	1.19 (1.05, 1.88)	−1.648	0.099
Lac/mmol/L	3.40 (1.70, 8.59)	3.00 (1.70, 6.80)	3.50 (1.70, 9.90)	−1.084	0.279
SO_2_/%	98.60 (95, 99)	98 (95, 99)	98.75 (95, 99)	−0.340	0.734
White blood cell count/ × 10^9^/L	14.75 ± 6.58	15.22 ± 6.99	14.65 ± 6.50	0.529	0.597
Albumin/g/L	31.97 ± 7.47	31.71 ± 7.16	32.03 ± 7.55	−0.256	0.798
Creatinine/μmol/L	112 (75, 211)	85 (68, 123)	126 (78, 226)	−3.043	0.002
Prothrombin time/s	15.30 (13.10, 18.20)	13.80 (11.80, 16.50)	15.65 (13.50, 18.60)	−2.798	0.005
NSE/ng/ml	36.50 (18.90, 115.10)	18.90 (12.60, 55.40)	55.35 (21.20, 136.80)	−4.669	< 0.001
S-100β/ug/L	0.58 (0.13, 1.52)	0.09 (0.04, 0.69)	0.97 (0.25, 2.00)	−4.188	< 0.001

This table summarizes clinical data obtained within 72 h after cardiac arrest.

### 3.2 EEG features and prognosis of patients

Table 2 compares the EEG features of patients at different time windows (Day 1, Day 2–5, >Day 5) after CA. Patients with abnormal background amplitude, background dominant frequency in the slow band (δ/θ) or no obvious EEG activity, discontinuous background, the absence of reactivity, and the absence of sleep waveforms had higher rates of poor prognosis at Day 1, Day 2–5, and >Day 5 compared with their respective control groups (all *P* < 0.001).

**Table 2:** Relationship between EEG features and prognosis at different time windows.

EEG results		Day 1				Day 2-Day 5				>**Day 5**			
		*N*	Good	Poor	*P*	*N*	Good	Poor	*P*	*N*	Good	Poor	*P*
**Overall**	**119**	**15.97 (19)**	**84.03 (100)**		**127**	**17.32 (22)**	**82.68 (105)**		**98**	**23.47 (24)**	**76.53 (75)**		
Background amplitude	Normal	42	18 (42.86)	24 (57.14)	< 0.001	58	21 (36.21)	37 (63.79)	< 0.001	61	23 (37.70)	38 (62.30)	< 0.001
	Abnormal	77	1 (1.30)	76 (98.70)		69	1 (1.45)	68 (98.55)		37	0 (0.00)	37 (100.00)	
Background dominant frequency	Fast frequency band (α/β)	24	10 (41.67)	14 (58.33)	< 0.001	27	14 (51.85)	13 (48.15)	< 0.001	24	15 (62.50)	9 (37.50)	< 0.001
	Slow frequency band (θ/δ)/Absence of prominent EEG activity	95	9 (9.47)	86 (90.53)		100	8 (8.00)	92 (92.00)		74	8 (10.81)	66 (89.19)	
Background continuity	Continuous	45	18 (40.00)	27 (60.00)	< 0.001	58	20 (34.48)	38 (65.52)	< 0.001	64	23 (35.94)	41 (64.06)	< 0.001
	Discontinuous	74	1 (1.35)	73 (98.65)		69	2 (2.90)	67 (97.10)		34	0 (0.00)	34 (100.00)	
Background symmetry	Markedly asymmetric	5	2 (40.00)	3 (60.00)	0.202	3	2 (66.67)	1 (33.33)	0.057	5	2 (40.00)	3 (60.00)	0.576
	Mildly asymmetric	4	1 (25.00)	3 (75.00)		4	1 (25.00)	3 (75.00)		4	1 (25.00)	3 (75.00)	
	Symmetric	110	16 (14.55)	94 (85.45)		120	19 (15.83)	101 (84.17)		89	20 (22.47)	69 (77.53)	
Reactivity	Present	44	18 (40.91)	26 (59.09)	< 0.001	56	20 (35.71)	36 (64.29)	< 0.001	62	23 (37.10)	39 (62.90)	< 0.001
	Absent	75	1 (1.33)	74 (98.67)		71	2 (2.82)	69 (97.18)		36	0 (0.00)	36 (100.00)	
SIRPID	Absent	117	19 (16.24)	98 (83.76)	0.999	122	22 (18.03)	100 (81.97)	0.659	92	23 (25.00)	69 (75.00)	0.331
	Present	2	0 (0.00)	2 (100.00)		5	0 (0.00)	5 (100.00)		6	0 (0.00)	6 (100.00)	
Epileptiform discharges	Absent	84	16 (19.05)	68 (80.95)	0.422	101	17 (16.83)	84 (83.17)	0.339	82	21 (25.61)	61 (74.39)	0.678
	Periodic discharges	3	0 (0.00)	3 (100.00)		7	0 (0.00)	7 (100.00)		4	0 (0.00)	4 (100.00)	
	Spikes and slow waves or sharp and slow waves	32	3 (9.38)	29 (90.63)		19	5 (26.32)	14 (73.68)		12	2 (16.67)	10 (83.33)	
Sleep waveforms	Present	10	6 (60.00)	4 (40.00)	< 0.001	16	7 (43.75)	9 (56.25)	0.008	12	8 (66.67)	4 (33.33)	< 0.001
	Absent	109	13 (11.93)	96 (88.07)		111	15 (13.51)	96 (86.49)		86	15 (17.44)	71 (82.56)	

Fisher's exact test without statistical measure.

### 3.3 Prediction of poor prognosis by the SERS

**Figure 2:**
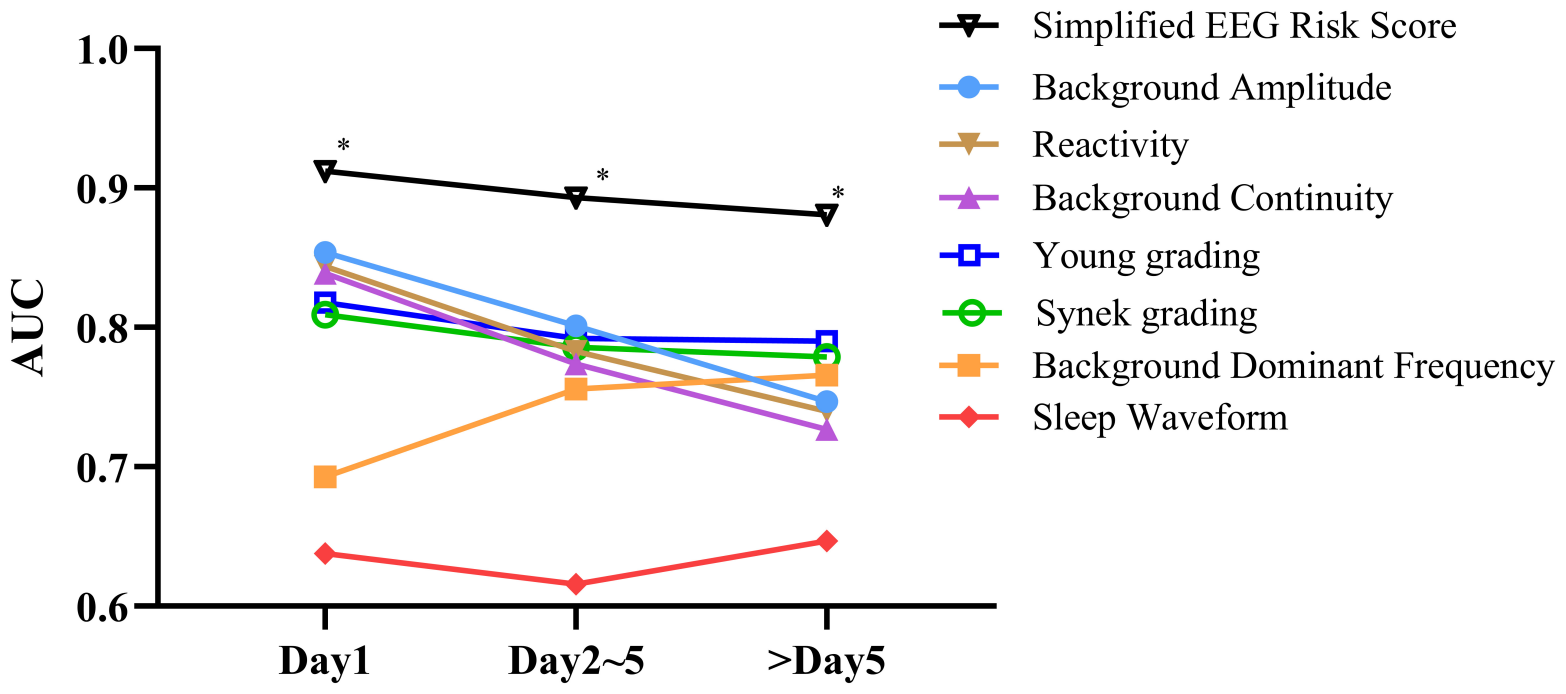
AUC values for predicting poor prognosis using SERS and Single EEG Features and Common EEG Scores (*The AUC values for the SERS are higher than that of single EEG features and common EEG scores within the same time window, *P* < 0.05).

**Figure 3:**
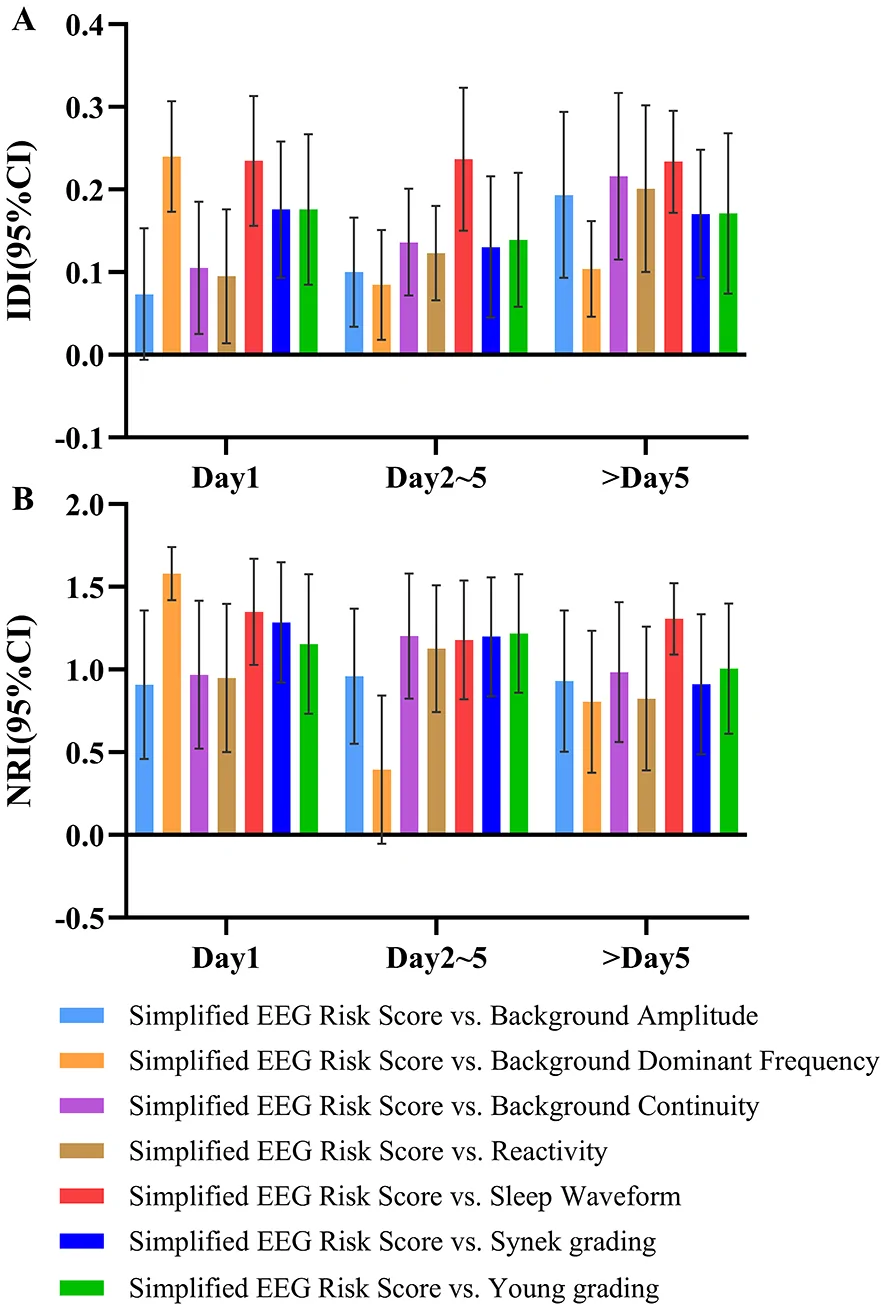
Comparison of the predictive performance for poor prognosis between the SERS and single EEG features and common EEG scores.

Table 3 presents the scoring results of the SERS for predicting poor prognosis. Based on the β values from the univariate logistic regression model, individual EEG features were assigned scores, and the sum of these scores constituted the SERS. The SERS demonstrated higher AUC values for predicting poor prognosis on Day 1, Day 2–5, and >Day 5 after CA compared with single EEG features, Synek grading, and Young grading (see Figure 2). The results of IDI and NRI indicated that the SERS had better predictive performance (see Figure 3). Additionally, the AUC values for predicting prognosis based on background amplitude and continuity gradually decreased on Day 1, Day 2–5, and >Day 5 after CA. In contrast, the AUC values for reactivity and sleep patterns first decreased and then increased, while the AUC value for dominant frequency gradually rose. The total score of the SERS showed minimal change in AUC values across these time periods (see Figure 2). Specific numerical values for the AUC, IDI, and NRI of each EEG feature are provided in Supplemental Tables 2, 3.

**Table 3:** SERS assignment table.

Variable	Univariate logistic regression model			Scoring assignment			
	β	*P*	OR (95%CI)	Category	Score	Category	Score
Day 1							
Background amplitude	4.043	< 0.001	56.989 (7.226, 449.444)	Normal	0	Abnormal	4
Background dominant frequency	1.921	< 0.001	6.825 (2.357, 19.761)	Fast frequency band (α/β)	0	Slow frequency band (θ/δ)/Absence of prominent EEG activity	2
Background continuity	3.885	< 0.001	48.659 (6.193, 382.312)	Continuous	0	Discontinuous	4
Reactivity	3.936	< 0.001	51.222 (6.512, 402.909)	Present	0	Absent	4
Sleep waveform	2.405	0.001	11.077 (2.755, 44.538)	Present	0	Absent	2.5
Day 2 ~ 5							
Background amplitude	3.653	< 0.001	38.589 (4.990, 298.422)	Normal	0	Abnormal	4
Background dominant frequency	2.516	< 0.001	12.382 (4.355, 35.204)	Fast frequency band	0	Slow frequency band (θ/δ)/Absence of prominent EEG activity	2.5
Background continuity	2.870	< 0.001	17.632 (3.906, 79.581)	Continuous	0	Discontinuous	3
Reactivity	2.953	< 0.001	19.167 (4.241, 86.623)	Present	0	Absent	3
Sleep waveform	1.605	0.005	4.978 (1.612, 15.375)	Present	0	Absent	1.5
>Day 5							
Background amplitude	3.824	0.009	45.795 (2.583, 811.833)	Normal	0	Abnormal	4
Background dominant frequency	2.547	< 0.001	12.763 (4.276, 38.099)	Fast frequency band	0	Slow frequency band (θ/δ)/Absence of prominent EEG activity	2.5
Background continuity	3.666	0.013	39.089 (2.197, 695.465)	Continuous	0	Discontinuous	4
Reactivity	3.772	0.010	43.447 (2.448, 771.070)	Present	0	Absent	4
Sleep waveform	2.165	0.001	8.714 (2.348, 32.338)	Present	0	Absent	2.5

### 3.4 Multivariate analysis results of poor prognosis

Table 4 shows the results of multivariate logistic regression analysis for general clinical indicators, clinical biochemical indicators, and EEG features associated with poor prognosis. In Model 1, which included general clinical indicators, initial rhythm (OR = 0.289, 95%CI = 0.127–0.653), APACHE II score (OR = 1.067, 95%CI = 1.017–1.119), and corneal reflex (OR = 5.151, 95%CI = 2.041–12.995) were identified as predictors of poor prognosis (all *P* < 0.05) after stepwise selection. In Model 2, which included clinical biochemical indicators, NSE (OR = 1.014, 95%CI = 1.006–1.022) was identified as a predictor of poor prognosis on the basis of stepwise selection. Results from Models 3 to 5 showed that the SERS on Day 1 (OR = 1.402, 95%CI = 1.128–1.744), Day 2–5 (OR = 1.484, 95%CI = 1.190–1.850), and >Day 5 (OR = 1.800, 95%CI = 1.300–2.494) were independent predictors of poor prognosis after adjusting for initial rhythm, APACHE II score, corneal reflex, and NSE.

**Table 4:** Multivariable logistic regression results for predicting poor prognosis.

Variable	β	Standard error	Wald χ2	*P*	OR (95%CI)
Model 1^a^					
Initial rhythm					1.0 (reference)
Non-ventricular fibrillation					
Ventricular fibrillation	−1.243	0.417	8.886	0.003	0.289 (0.127, 0.653)
APACHE II Score	0.065	0.025	6.996	0.008	1.067 (1.017, 1.119)
Pupillary reflex					
Absent	1.639	0.472	12.051	0.001	5.151 (2.041, 12.995)
Present					1.0 (reference)
Model 2^b^					
NSE/ng/ml	0.014	0.004	11.154	0.001	1.014 (1.006, 1.022)
Model 3^c^					
Day 1 SERS	0.338	0.111	9.247	0.002	1.402 (1.128, 1.744)
Model 4^d^					
Day 2 ~ 5 SERS	0.395	0.112	12.31	< 0.001	1.484 (1.190, 1.850)
Model 5^e^					
>Day 5 SERS	0.588	0.166	12.522	< 0.001	1.800 (1.300, 2.494)

^a^Sample size: 251, variables included: age, initial rhythm, CCI score, GCS score, APACHE II score, pupillary reflex, CT/MRI results, stepwise variable selection.

^b^Sample size: 251, variables included: creatine kinase, creatine time, NSE, S-100β, stepwise variable selection.

^c^Sample size: 119, adjusted variables: initial rhythm, APACHE II score, pupillary reflex, NSE.

^d^Sample size: 127, adjusted variables: initial rhythm, APACHE II score, pupillary reflex, NSE.

^e^Sample size: 98, adjusted variables: initial rhythm, APACHE II score, pupillary reflex, NSE.

### 3.5 Construction and performance evaluation of predictive models

**Figure 4:**
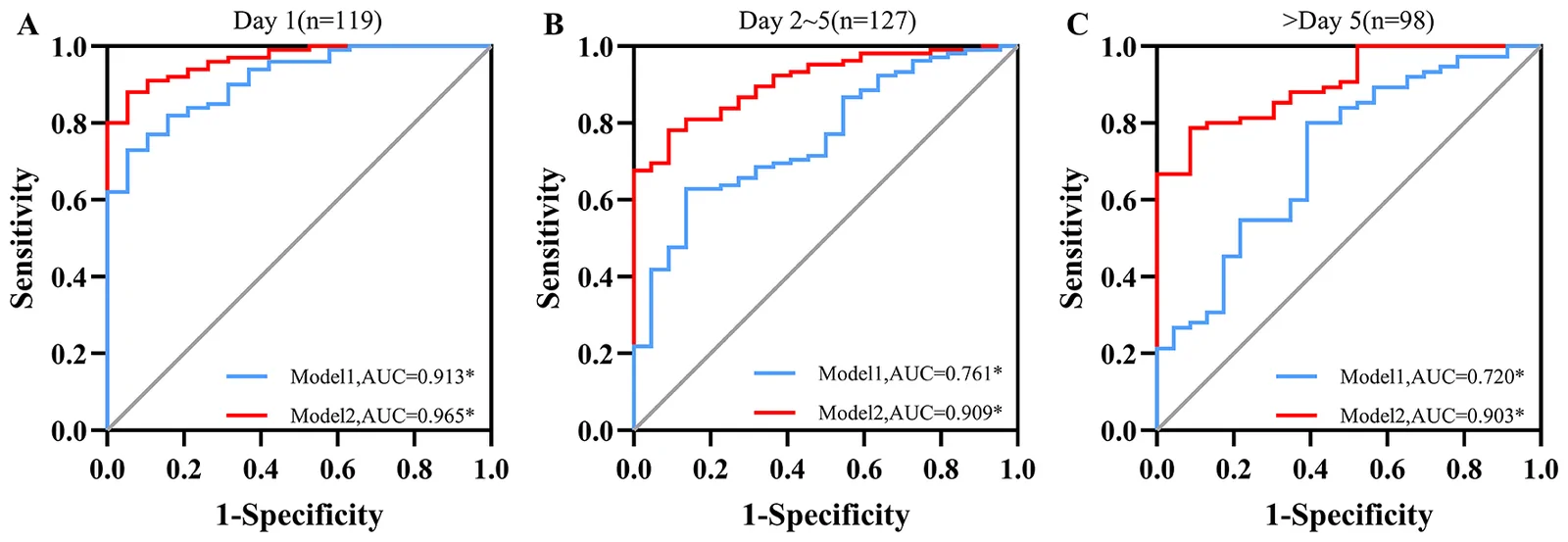
ROC curves of model predicting poor prognosis (*P* < 0.05).

Compared with Model 1, which was based on general clinical characteristics and clinical biochemical indicators for predicting poor prognosis in comatose patients after CA, Model 2, which included the SERS, had higher AUC values (Day 1 AUC: 0.965 vs 0.913; Day 2–5 AUC: 0.909 vs 0.761; >Day 5 AUC: 0.903 vs 0.720, see Table 5 and Figure 4). The results of IDI and NRI showed that Model 2 had higher predictive performance than Model 1 in all three time windows (Day 1, Day 2–5, >Day 5, see Supplemental Table 4). Internal validation was performed using leave-one-out cross-validation, with accuracy, sensitivity, and specificity for predicting poor prognosis on Day 1 after CA being 89.08%, 88.00%, and 94.74%, respectively; on Day 2–5, they were 80.32%, 78.10%, and 90.91%, respectively; and on >Day 5, they were 81.63%, 78.67%, and 91.30%, respectively (see Table 5).

**Table 5:** Construction and performance evaluation of different prediction models.

Model	cut-off value	Accuracy^a^	Sensitivity^a^	Specificity^a^	AUC (95% CI)	Z	*P*
Day 1 (*n* = 119)							
Model 1	0.920	76.47%	73.00%	94.74%	0.913 (0.854, 0.971)	13.775	< 0.001
Model 2	0.857	89.08%	88.00%	94.74%	0.965 (0.934, 0.995)	29.643	< 0.001
Day 2–Day 5 (*n* = 127)							
Model 1	0.847	66.93%	62.86%	86.36%	0.761 (0.659, 0.863)	5.018	< 0.001
Model 2	0.797	80.32%	78.10%	90.91%	0.909 (0.855, 0.963)	14.872	< 0.001
>Day 5 (*n* = 98)							
Model 1	0.719	75.51%	80.00%	60.87%	0.720 (0.597, 0.843)	3.512	< 0.001
Model 2	0.764	81.63%	78.67%	91.30%	0.903 (0.842, 0.963)	13.126	< 0.001

Model 1 included initial rhythm, APACHE II score, pupillary reflex, and NSE.

Model 2 included the SERS based on Model 1.

^a^Results from leave-one-out cross-validation.

### 3.6 Risk stratification using the SERS

Supplemental Table 5 shows the relationship between risk stratification based on the SERS and poor prognosis. On Day 1 after CA, a score of 0 indicated low risk, 2–8.5 indicated medium risk, and 10–16.5 indicated high risk, with poor prognosis rates of 33.33%, 59.46%, and 100%, respectively. On Day 2–5, a score of 0 indicated low risk, 1.5–7 indicated medium risk, and 7.5–14 indicated high risk, with poor prognosis rates of 20.00%, 67.35%, and 97.26%, respectively. On >Day 5, a score of 0 indicated low risk, 2.5–6.5 indicated medium risk, and 8–17 indicated high risk, with poor prognosis rates of 11.11%, 65.97%, and 100%, respectively.

## 4 Discussion

This study included 251 comatose patients after CA to investigate the relationship between EEG features and prognosis and their predictive value. The results showed that EEG features such as abnormal background amplitude, background dominant frequency in the slow band (δ/θ), discontinuous background, the absence of reactivity, and the absence of sleep waveforms were significantly associated with poor prognosis (*P* < 0.001). Based on these EEG features, we developed a SERS and evaluated its predictive performance in different time windows after CA. The score demonstrated significantly higher predictive efficacy than single EEG features, common EEG scores, and traditional clinical indicators in all three time windows (Day 1, Day 2–5, >Day 5), with AUC values of 0.963, 0.954, and 0.944, respectively. It also exhibited high predictive accuracy (89.08%, 80.32%, 81.63%), sensitivity (88.00%, 78.10%, 78.67%), and specificity (94.74%, 90.91%, 91.30%). The SERS has high predictive performance and cross-time stability. Moreover, it can accurately stratify the risk of poor prognosis in comatose patients after CA, making it a reliable tool for predicting neurological outcomes and providing strong support for dynamic prognostic assessment in clinical practice.

EEG features hold significant value in predicting the prognosis of comatose patients after CA. For instance, continuous suppression of background continuity and disappearance of EEG reactivity usually indicate poor prognosis (25). However, studies by Ruijter et al. and Grippo et al. have shown that when used as single predictive indicators, the false-positive rates of EEG background continuity and EEG reactivity are as high as 37.6% and 58.3%, respectively (26, 27). This indicates that the predictive ability of single EEG features may be limited because they cannot comprehensively reflect brain function status and capture the complexity of brain function changes. Common EEG scores, such as Synek grading and Young grading, incorporate important EEG features like background frequency, EEG reactivity, and epileptiform discharges. However, in clinical practice, we have found that due to the lack of inclusion of key EEG features such as sleep, their clinical application has certain limitations. Therefore, the SERS developed in this study integrated key EEG features such as background amplitude, background dominant frequency, background continuity, reactivity, epileptiform discharges, and sleep waveforms. This multidimensional feature integration method can more comprehensively reflect brain function status and accurately assess the severity of brain injury. The results showed that the SERS has higher accuracy in assessing brain function status, with significantly higher predictive efficacy than single EEG features and common EEG scores. Compared with traditional predictive models based on general clinical characteristics and clinical biochemical indicators, the SERS demonstrated higher predictive efficacy with significantly higher AUC values, enabling more accurate identification of patients with poor prognosis and providing stronger decision-making support for clinicians to optimize treatment plans.

Previous studies have confirmed that the predictive value of EEG features has significant timeliness (28). A study by Barry J. Ruijter et al. showed that when EEG showed discontinuous background 6 h after CA, the probability of predicting a good outcome was 80%. However, this probability gradually decreased over time, dropping to 0% by 120 h (26). Additionally, a prospective cohort study found that among 104 unresponsive patients, 16 patients was detected as “brain activation” by EEG at a median of 4 days after injury (29). It was observed from EEG recordings that “brain activation” is associated with a good neurological outcome at 12 months. Moreover, the results of this study showed that in the three time windows (Day 1, Day 2–5, >Day 5) after CA, the AUC values of background amplitude and continuity gradually decreased, while the predictive efficacy of reactivity and sleep waveforms showed a trend of first decreasing and then increasing, and the AUC value of background frequency gradually increased. Therefore, for prognostic prediction of comatose patients after CA, it is important to establish a comprehensive EEG score that performs well in multiple time windows. This study quantified the β values of EEG features using univariate logistic regression to construct the SERS. This score broke through the traditional single-time-point evaluation framework, dividing the CA course into three stages (Day 1, Day 2–5, >Day 5) for dynamic score validation. The results showed that the score maintained robust predictive efficacy in all three time windows (AUC 0.84–0.89), retaining the biological interpretability of EEG features while optimizing predictive stability through mathematical modeling, providing a decision-making support tool with both timeliness and reliability for clinical practice.

## 5 Limitations

This study has some limitations. First, this was a single-center, retrospective study with a relatively small sample size, and no external validation was performed. The lack of validation in different medical settings limited its generalizability and reliability. Future studies should validate the effectiveness and reliability of this score in multicenter, larger populations. To this end, an online validation platform has been established for this study (website link: https://github.com/ShengYH2001/Simplified-EEG-Risk-Score), for researchers and clinicians to upload independent datasets to verify the performance of the SERS in different patient populations. Second, due to the retrospective study design and limitations in clinical data collection, some patients had missing EEG monitoring data in different time windows, leading to certain heterogeneity in the study samples across different time windows. It is worth noting that despite the differences in the sample population, the results of this study showed that the AUC values of the SERS in different time windows (Day 1, Day 2–5, >Day 5) after CA were all consistently above 0.88, indicating good cross-time stability. This preliminary result confirmed the clinical applicability of the SERS. However, to further strengthen the evidence, follow-up studies should conduct multicenter prospective research to collect continuous EEG data within unified time windows to systematically verify its stability and generalizability. Finally, although this study adjusted for some confounding factors through multivariate analysis, the treatment measures (such as the use of sedatives and analgesics, target temperature management) may affect EEG features, and these factors had not been completely ruled out. Future research should further optimize this score, consider more potential confounding factors, and combine artificial intelligence and machine learning techniques to further enhance its performance. In addition, withdrawal of life-sustaining therapy (WLST) may have influenced neurological outcomes and introduced a potential self-fulfilling prophecy bias. Although WLST decisions in our center were based on comprehensive clinical assessment rather than EEG findings alone, this influence could not be completely excluded because of the retrospective study design. To evaluate the potential impact of WLST, we performed a series of sensitivity analyses after excluding patients who underwent WLST (Supplemental Tables 6–9). The results demonstrated that the predictive performance of the SERS and the multivariable prediction models remained largely unchanged, with comparable AUC values and consistently superior performance of the SERS over individual EEG features and conventional EEG scores. These findings suggest that the main conclusions of this study are robust and are unlikely to be substantially affected by WLST. Nevertheless, future prospective multicenter studies incorporating standardized neuroprognostication protocols and predefined WLST decision-making processes are warranted to further validate our findings. Finally, we also recognize that this score was developed based on prolonged EEG recordings, and its predictive performance should not be directly extrapolated to routine short-duration EEG recordings; therefore, caution is needed when interpreting its applicability across different EEG monitoring modalities.

## 6 Conclusions

The SERS developed in this study not only demonstrates high predictive performance but also exhibits cross-time stability. It can accurately stratify the risk of poor prognosis in comatose patients after CA, providing a scientifically sound and practically useful evidence-based decision-making support for precise neurological assessment, individualized treatment timing selection, and rehabilitation strategy formulation in comatose patients after CA.

## Data Availability

The original contributions presented in the study are included in the article/Supplementary material, further inquiries can be directed to the corresponding author.
