# Supplementary material for: Prognostic assessment of comatose patients after cardiac arrest: construction of a simplified monitoring EEG risk score across different time windows
Source: Front Neurol. 2026 Aug 25;17:1812084. doi: 10.3389/fneur.2026.1812084 (PMC13549905; doi:10.3389/fneur.2026.1812084)
Supplement: Supplementary file 1 [file Table_1.doc]

**Supplemental Table 1 Standards for EEG Feature Classification**

| **EEG Feature Classification** | | **ACNS 2021 Standard Terminology and Definitions** |
| --- | --- | --- |
| Background Amplitude | Normal | Background activity amplitude within normal range (20-150μV) |
|  | Abnormal | Background activity amplitude outside normal range (≥150μV or <20μV) |
| Background Dominant Frequency | Fast frequency band  (α/β) | Background dominated by α waves (8-13Hz), β waves (>13Hz) |
|  | Slow frequency band  (θ/δ) /Absence of prominent EEG activity | Background dominated by θ waves (4-8Hz), δ waves (0.5-4Hz), or absence of prominent EEG activity |
| Background Continuity | Continuous | continuous, or with occasional (1-9% of the record) periods of attenuation (periods of lower voltage ≥10µV  but <50% of higher voltage background) or suppression (periods of lower voltage <10µV). |
|  | Discontinuous | Background activity with attenuation / suppression |
| Background Symmetry | markedly asymmetric | Amplitude difference ≥50% or frequency difference >1Hz |
|  | mildly asymmetric | Appropriate reference recording shows amplitude difference <50% or frequency difference between 0.5-1Hz |
|  | symmetric | No significant asymmetry observed |
| Reactivity | Present | Clear changes in EEG activity (frequency, amplitude, or form) after stimulation |
|  | Absent | No changes in EEG activity after stimulation |
| SIRPID | Absent | No rhythmic, periodic, or ictal discharges induced by stimulation |
|  | Present | Presence of rhythmic, periodic, or ictal discharges induced by stimulation (such as SI-RDA, SI-PDs, etc.) |
| epileptiform discharges | Absent | No spikes (<70ms), sharp waves (70-200ms), multiple spikes and waves, or slow waves accompanying them |
|  | periodic discharges | Periodic repeated spikes, consistent in form, clear intervals |
|  | spikes and slow waves or sharp and slow waves | Spikes, multiple spikes or sharp waves followed by slow waves appearing in a pattern of regular repetition |
| Sleep Waveforms | Present | Presence of normal sleep features (such as spindles, K-complexes) |
|  | Absent | Absence of normal sleep features |

**Supplemental Table 2 The AUC Values of The SERS and Single EEG Features and Common EEG Scores in Predicting Poor Prognosis**

| **Indicator** | **Day1(n=119)** | | **Day2-Day5(n=127)** | | **>Day5(n=98)** | |
| --- | --- | --- | --- | --- | --- | --- |
|  | **AUC(95%CI)** | **P** | **AUC(95%CI)** | **P** | **AUC(95%CI)** | **P** |
| Background Amplitude | 0.854(0.787,0.920) | <0.001 | 0.801(0.737,0.865) | <0.001 | 0.747(0.690,0.804) | <0.001 |
| Background Dominant Frequency | 0.693(0.573,0.813) | 0.002 | 0.756(0.649,0.864) | <0.001 | 0.766(0.660,0.872) | <0.001 |
| Background Continuity | 0.839(0.771,0.906 | <0.001 | 0.774(0.697,0.850) | <0.001 | 0.727(0.670,0.783) | <0.001 |
| Reactivity | 0.844(0.776,0.911) | <0.001 | 0.783(0.707,0.860) | <0.001 | 0.740(0.683,0.797) | <0.001 |
| Sleep Waveform | 0.638(0.529,0.747) | 0.013 | 0.616(0.513,0.719) | 0.027 | 0.647(0.544,0.750) | 0.005 |
| Synek grading | 0.809(0.712,0.906) | <0.001 | 0.786(0.686,0.887) | <0.001 | 0.779(0.670,0.889) | <0.001 |
| Young grading | 0.818(0.752,0.883) | <0.001 | 0.792(0.723,0.862) | <0.001 | 0.790(0.711,0.869) | <0.001 |
| SERS | 0.912(0.861,0.963) | <0.001 | 0.893(0.832,0.954) | <0.001 | 0.881(0.817,0.944) | <0.001 |

**Supplemental Table 3 Comparison of the Predictive Performance of SERS and Single EEG Features and Common EEG Scores in Predicting Poor Prognosis**

| **Indicator** | **AUC Difference (95%CI)^a^** | **P** | **IDI(95%CI)^a^** | **P** | **NRI(95%CI)^a^** | **P** |
| --- | --- | --- | --- | --- | --- | --- |
| Day1(n=119) |  |  |  |  |  |  |
| SERS vs. Background Amplitude | 0.058(0.004,0.113) | 0.037 | 0.073(-0.006,0.153) | 0.072 | 0.908(0.459,1.358) | <0.001 |
| SERS vs. Background Dominant Frequency | 0.219(0.116,0.322) | <0.001 | 0.240(0.173,0.307) | <0.001 | 1.580(1.420,1.740) | <0.001 |
| SERS vs. Background Continuity | 0.073(0.017,0.130) | 0.012 | 0.105(0.025,0.185) | 0.010 | 0.968(0.522,1.415) | <0.001 |
| SERS vs. Reactivity | 0.068(0.011,0.126) | 0.019 | 0.095(0.014,0.176) | 0.021 | 0.948(0.501,1.396) | <0.001 |
| SERS vs. Sleep Waveform | 0.274(0.182,0.367) | <0.001 | 0.235(0.156,0.313) | <0.001 | 1.349(1.029,1.670) | <0.001 |
| SERS vs. Synek grading | 0.103(0.011,0.195) | 0.028 | 0.176(0.093,0.258) | <0.001 | 1.284(0.921,1.648) | <0.001 |
| SERS vs. Young grading | 0.095(0.030,0.159) | 0.004 | 0.176(0.085,0.267) | <0.001 | 1.154(0.732,1.575) | <0.001 |
| Day2-Day5(n=127) |  |  |  |  |  |  |
| SERS vs. Background Amplitude | 0.092(0.035,0.149) | 0.002 | 0.100(0.034,0.166) | 0.003 | 0.959(0.552,1.367) | <0.001 |
| SERS vs. Background Dominant Frequency | 0.137(0.055,0.219) | 0.001 | 0.085(0.018,0.151) | 0.013 | 0.395(-0.054,0.843) | 0.092 |
| SERS vs. Background Continuity | 0.120(0.061,0.178) | <0.001 | 0.136(0.072,0.201) | <0.001 | 1.203(0.824,1.581) | <0.001 |
| SERS vs. Reactivity | 0.110(0.055,0.165) | <0.001 | 0.123(0.066,0.180) | <0.001 | 1.126(0.743,1.510) | <0.001 |
| SERS vs. Sleep Waveform | 0.277(0.169,0.385) | <0.001 | 0.237(0.150,0.323) | <0.001 | 1.179(0.819,1.539) | <0.001 |
| SERS vs. Synek grading | 0.092(-0.008,0.191) | 0.072 | 0.111(0.024,0.198) | 0.012 | 1.198(0.839,1.557) | <0.001 |
| SERS vs. Young grading | 0.101(0.025,0.176) | 0.009 | 0.139(0.058,0.220) | 0.001 | 1.217(0.859,1.575) | <0.001 |
| >Day5(n=98) |  |  |  |  |  |  |
| SERS vs. Background Amplitude | 0.134(0.069,0.199) | <0.001 | 0.193(0.093,0.294) | <0.001 | 0.931(0.504,1.358) | <0.001 |
| SERS vs. Background Dominant Frequency | 0.115(0.051,0.178) | <0.001 | 0.104(0.046,0.162) | <0.001 | 0.805(0.376,1.234) | 0.001 |
| SERS vs. Background Continuity | 0.154(0.088,0.220) | <0.001 | 0.216(0.115,0.317) | <0.001 | 0.984(0.561,1.408) | <0.001 |
| SERS vs. Reactivity | 0.141(0.075,0.206) | <0.001 | 0.201(0.100,0.302) | <0.001 | 0.824(0.390,1.259) | 0.001 |
| SERS vs. Sleep Waveform | 0.233(0.153,0.314) | <0.001 | 0.234(0.172,0.295) | <0.001 | 1.307(1.091,1.522) | <0.001 |
| SERS vs. Synek grading | 0.053(-0.024,0.130) | 0.179 | 0.107(0.027,0.188) | 0.009 | 0.798(0.361,1.234) | 0.001 |
| SERS vs. Young grading | 0.090(0.014,0.166) | 0.020 | 0.171(0.074,0.268) | 0.001 | 1.005(0.612,1.399) | <0.001 |
| ^a^ SERS vs. Single EEG Features or Common EEG Scores | | | | | | |

| **Supplemental Table 4 Comparisons of Prediction Results for Poor Prognosis Using Different Models** | | | | | | |
| --- | --- | --- | --- | --- | --- | --- |
| **Indicator** | **AUC Difference (95%CI)^a^** | **P** | **IDI(95%CI)^a^** | **P** | **NRI(95%CI)^a^** | **P** |
| Day1(n=119) |  |  |  |  |  |  |
| Model 2 vs. Model 1 | 0.049(0.009,0.089) | 0.016 | 0.309(0.229,0.388) | <0.001 | 1.529(1.224,1.835) | <0.001 |
| Day2-Day5(n=127) |  |  |  |  |  |  |
| Model 2 vs. Model 1 | 0.143(0.054,0.233) | 0.002 | 0.189(0.096,0.282) | <0.001 | 1.031(0.643,1.419) | <0.001 |
| >Day5(n=98) |  |  |  |  |  |  |
| Model 2 vs. Model 1 | 0.181(0.074,0.288) | 0.001 | 0.275(0.167,0.382) | <0.001 | 1.226(0.864,1.587) | <0.001 |
| Model 1 included initial rhythm, APACHE II score, pupillary reflex, NSE. | | | | | | |
| Model 2 included the SERS based on Model 1. | | | |  |  |  |

**Supplemental Table 5 Risk Stratification Using the SERS**

| ***Day1 (n=119)*** | | | | | | | | | | | | | | | ***Day2-Day5(n=127)*** | | | | | | | | | | | | | | | | ***>Day5(n=98)*** | | | | | | | | | | | | |
| --- | --- | --- | --- | --- | --- | --- | --- | --- | --- | --- | --- | --- | --- | --- | --- | --- | --- | --- | --- | --- | --- | --- | --- | --- | --- | --- | --- | --- | --- | --- | --- | --- | --- | --- | --- | --- | --- | --- | --- | --- | --- | --- | --- |
| **Total EEG Score^a^** | | **Risk of Poor Prognosis^b^** | **Stratification^c^** | | | **Actual Poor Prognosis Rate** | | |  | | **Total EEG Score^a^** | | | **Risk of Poor Prognosis^b^** | | | | **Stratification^c^** | | | **Actual Poor Prognosis Rate** | | |  | **Total EEG Score^a^** | | | | **Risk of Poor Prognosis^b^** | | | | | **Stratification^c^** | | **Actual Poor Prognosis Rate** | | | |  | | | |
| 0 | | 0.273 | Low Risk | | | 33.33% | | |  | | 0 | | | 0.338 | | | | Low Risk | | | 20.00% | | |  | 0 | | | | 0.184 | | | | | Low Risk | | 11.11% | | | |  | | | |
| 2 | | 0.433 | Medium Risk | | | 59.46% | | |  | | 1.5 | | | 0.485 | | | | Medium Risk | | | 67.35% | | |  | 2.5 | | | | 0.464 | | | | | Medium Risk | | 65.91% | | | |  | | | |
| 2.5 | | 0.476 | Medium Risk | | |  |  |  |  | | 2.5 | | | 0.586 | | | | Medium Risk | | |  |  |  |  | 4 | | | | 0.659 | | | | | Medium Risk | |  |  |  |  |  | | | |
| 4 | | 0.607 | Medium Risk | | |  |  |  |  | | 3 | | | 0.634 | | | | Medium Risk | | |  |  |  |  | 5 | | | | 0.768 | | | | | Medium Risk | |  |  |  |  |  | | | |
| 4.5 | | 0.648 | Medium Risk | | |  |  |  |  | | 4 | | | 0.722 | | | | Medium Risk | | |  |  |  |  | 6.5 | | | | 0.881 | | | | | Medium Risk | |  |  |  |  |  | | | |
| 6 | | 0.758 | Medium Risk | | |  |  |  |  | | 4.5 | | | 0.761 | | | | Medium Risk | | |  |  |  |  | 8 | | | | 0.943 | | | | | High Risk | | 100.00% | | | |  | | | |
| 6.5 | | 0.789 | Medium Risk | | |  |  |  |  | | 5.5 | | | 0.827 | | | | Medium Risk | | |  |  |  |  | 9 | | | | 0.966 | | | | | High Risk | |  |  |  |  |  | | | |
| 8 | | 0.864 | Medium Risk | | |  |  |  |  | | 6 | | | 0.855 | | | | Medium Risk | | |  |  |  |  | 10.5 | | | | 0.984 | | | | | High Risk | |  |  |  |  |  | | | |
| 8.5 | | 0.884 | Medium Risk | | |  |  |  |  | | 6.5 | | | 0.878 | | | | Medium Risk | | |  |  |  |  | 12 | | | | 0.993 | | | | | High Risk | |  |  |  |  |  | | | |
| 10 | | 0.928 | High Risk | | | 100.00% | | |  | | 7 | | | 0.898 | | | | Medium Risk | | |  |  |  |  | 13 | | | | 0.996 | | | | | High Risk | |  |  |  |  |  | | | |
| 10.5 | | 0.939 | High Risk | | |  |  |  |  | | 7.5 | | | 0.915 | | | | High Risk | | | 97.26% | | |  | 14.5 | | | | 0.998 | | | | | High Risk | |  |  |  |  |  | | | |
| 12 | | 0.963 | High Risk | | |  |  |  |  | | 8 | | | 0.930 | | | | High Risk | | |  |  |  |  | 17 | | | | 1.000 | | | | | High Risk | |  |  |  |  |  | | | |
| 12.5 | | 0.969 | High Risk | | |  |  |  |  | | 8.5 | | | 0.942 | | | | High Risk | | |  | | | | | |  |  | | | | |  | | | |  | |  | | |  | |
| 14 | | 0.981 | High Risk | | |  |  |  |  | | 9.5 | | | 0.961 | | | | High Risk | | |  |  |  |  |  |  |  |  | | | | |  | | | |  | |  | | |  | |
| 14.5 | | 0.984 | High Risk | | |  |  |  |  | | 10 | | | 0.968 | | | | High Risk | | |  |  |  |  |  |  |  |  | | | | |  | | | |  | |  | | |  | |
| 16.5 | | 0.992 | High Risk | | |  |  |  |  | | 10.5 | | | 0.973 | | | | High Risk | | |  |  |  |  |  |  |  |  | | | | |  | | | |  | |  | | |  | |
|  |  | | |  |  | | | | | | |  | 11 | | | | 0.978 | | | High Risk | |  | | | | | | | |  | |  | | |  | | |  | | |  | |  |
|  |  | | |  |  | | | | | | |  | 11.5 | | | | 0.982 | | | High Risk | |  | | | | | | | |  | |  | | |  | | |  | | |  | |  |
|  |  | | |  |  | | | | | | |  | 12.5 | | | | 0.988 | | | High Risk | |  | | | | | | | |  | |  | | |  | | |  | | |  | |  |
|  |  | | |  |  | | | | | | |  | 13 | | | | 0.990 | | | High Risk | |  | | | | | | | |  | |  | | |  | | |  | | |  | |  |
|  |  | | |  |  | | | | | | |  | 14 | | | | 0.993 | | | High Risk | |  | | | | | | | |  | |  | | |  | | |  | | |  | |  |
| ^a^ Combination of all scores. | | | | | | |  |  | |  | | | | |  |  | | |  | | | |  | | |  | | | |  | |  | | |  | | |  | | |  | |  |
| ^b^ Based on logistic regression model. | | | | | | |  |  | |  | | | | |  |  | | |  | | | |  | | |  | | | |  | |  | | |  | | |  | | |  | |  |
| ^c^ <0.5 is low risk, 0.4-0.9 is medium risk, ≥0.9 is high risk. | | | | | | | | | | | | | | |  |  | | |  | | | |  | | |  | | | |  | |  | | |  | | |  | | |  | |  |

**Supplemental Table 6 AUC Values of the SERS, Individual EEG Features, and Conventional EEG Scores for Predicting Poor Neurological Outcome After Exclusion of Patients Undergoing WLST**

| **Indicator** | **Day1(n=119)** | | **Day2-Day5(n=127)** | | **>Day5(n=98)** | |
| --- | --- | --- | --- | --- | --- | --- |
|  | **AUC(95%CI)** | **P** | **AUC(95%CI)** | **P** | **AUC(95%CI)** | **P** |
| Background Amplitude | 0.857(0.786,0.928) | <0.001 | 0.782(0.713,0.851) | <0.001 | 0.726(0.663,0.788) | <0.001 |
| Background Dominant Frequency | 0.702(0.580,0.823) | 0.001 | 0.757(0.648,0.866) | <0.001 | 0.762(0.654,0.870) | <0.001 |
| Background Continuity | 0.837(0.764,0.910) | <0.001 | 0.753(0.672,0.835) | <0.001 | 0.710(0.648,0.772) | <0.001 |
| Reactivity | 0.830(0.756,0.903) | <0.001 | 0.766(0.684,0.847) | <0.001 | 0.718(0.656,0.780) | <0.001 |
| Sleep Waveform | 0.630(0.520,0.741) | 0.021 | 0.610(0.506,0.715) | 0.039 | 0.650(0.547,0.753) | 0.004 |
| Synek grading | 0.804(0.699,0.909) | <0.001 | 0.779(0.672,0.886) | <0.001 | 0.756(0.637,0.874) | <0.001 |
| Young grading | 0.806(0.730,0.881) | <0.001 | 0.774(0.696,0.853) | <0.001 | 0.777(0.690,0.864) | <0.001 |
| SERS | 0.909(0.852,0.966) | <0.001 | 0.888(0.821,0.955) | <0.001 | 0.874(0.806,0.943) | <0.001 |

**Supplemental Table 7 Comparison of the Predictive Performance of the SERS, Individual EEG Features, and Conventional EEG Scores for Predicting Poor Neurological Outcome After Excluding Patients Who Underwent WLST**

| **Indicator** | | **AUC Difference (95%CI)^a^** | | **P** | | **IDI(95%CI)^a^** | | **P** | | **NRI(95%CI)^a^** | | **P** | | |
| --- | --- | --- | --- | --- | --- | --- | --- | --- | --- | --- | --- | --- | --- | --- |
| Day1(n=92) | |  | |  | |  | |  | |  | |  | | |
| SERS vs. Background Amplitude | | 0.052(-0.007,0.110) | | 0.082 | | 0.060(-0.024,0.143) | | 0.161 | | 0.820(0.355,1.286) | | 0.001 | | |
| SERS vs. Background Dominant Frequency | | 0.208(0.100,0.315) | | <0.001 | | 0.249(0.170,0.328) | | <0.001 | | 1.562(1.372,1.751) | | <0.001 | | |
| SERS vs. Background Continuity | | 0.073(0.010,0.135) | | 0.023 | | 0.107(0.023,0.192) | | 0.013 | | 0.903(0.442,1.364) | | <0.001 | | |
| SERS vs. Reactivity | | 0.079(0.016,0.143) | | 0.014 | | 0.122(0.037,0.207) | | 0.005 | | 0.930(0.471,1.389) | | <0.001 | | |
| SERS vs. Sleep Waveform | | 0.279(0.185,0.372) | | <0.001 | | 0.297(0.215,0.378) | | <0.001 | | 1.534(1.340,1.728) | | <0.001 | | |
| SERS vs. Synek grading | | 0.105(0.005,0.205) | | 0.039 | | 0.192(0.097,0.287) | | <0.001 | | 1.301(0.926,1.675) | | <0.001 | | |
| SERS vs. Young grading | | 0.104(0.029,0.178) | | 0.006 | | 0.199(0.100,0.299) | | <0.001 | | 1.145(0.714,1.576) | | <0.001 | | |
| Day2-Day5(n=104) | |  | |  | |  | |  | |  | |  | | |
| SERS vs. Background Amplitude | | 0.106(0.043,0.168) | | 0.001 | | 0.115(0.045,0.185) | | 0.001 | | 0.942(0.525,1.360) | | <0.001 | | |
| SERS vs. Background Dominant Frequency | | 0.131(0.047,0.215) | | 0.002 | | 0.079(0.002,0.155) | | 0.043 | | 0.348(-0.111,0.808) | | 0.147 | | |
| SERS vs. Background Continuity | | 0.135(0.072,0.197) | | <0.001 | | 0.155(0.087,0.223) | | <0.001 | | 1.180(0.791,1.568) | | <0.001 | | |
| SERS vs. Reactivity | | 0.123(0.063,0.182) | | <0.001 | | 0.138(0.078,0.199) | | <0.001 | | 1.082(0.687,1.477) | | <0.001 | | |
| SERS vs. Sleep Waveform | | 0.278(0.167,0.389) | | <0.001 | | 0.258(0.166,0.349) | | <0.001 | | 1.149(0.777,1.521) | | <0.001 | | |
| SERS vs. Synek grading | | 0.109(-0.004,0.222) | | 0.059 | | 0.135(0.040,0.230) | | 0.006 | | 1.173(0.803,1.543) | | <0.001 | | |
| SERS vs. Young grading | | 0.114(0.027,0.200) | | 0.010 | | 0.153(0.064,0.241) | | 0.001 | | 1.173(0.803,1.543) | | <0.001 | | |
| >Day5(n=85) | |  | |  | |  | |  | |  | |  | | |
| SERS vs. Background Amplitude | | 0.149(0.078,0.220) | | <0.001 | | 0.205(0.101,0.308) | | <0.001 | | 0.950(0.516,1.383) | | <0.001 | | |
| SERS vs. Background Dominant Frequency | | 0.113(0.050,0.176) | | <0.001 | | 0.113(0.053,0.173) | | <0.001 | | 0.746(0.304,1.188) | | 0.002 | | |
| SERS vs. Background Continuity | | 0.165(0.093,0.237) | | <0.001 | | 0.223(0.119,0.327) | | <0.001 | | 0.982(0.552,1.412) | | <0.001 | | |
| SERS vs. Reactivity | | 0.157(0.085,0.228) | | <0.001 | | 0.214(0.109,0.319) | | <0.001 | | 0.788(0.342,1.234) | | 0.001 | | |
| SERS vs. Sleep Waveform | | 0.225(0.143,0.307) | | <0.001 | | 0.230(0.161,0.299) | | <0.001 | | 1.226(0.983,1.468) | | <0.001 | | |
| SERS vs. Synek grading | | 0.119(0.017,0.221) | | 0.022 | | 0.198(0.117,0.280) | | <0.001 | | 0.907(0.475,1.340) | | <0.001 | | |
| SERS vs. Young grading | | 0.098(0.016,0.179) | | 0.019 | | 0.179(0.080,0.278) | | <0.001 | | 0.985(0.579,1.390) | | <0.001 | | |
| ^a^ SERS vs. Single EEG Features or Common EEG Scores **Supplemental Table 8 Construction and Performance Evaluation of Different Prediction Models After Excluding Patients Who Underwent WLST** **Model** \| **cut-off value** \| **Accuracy^a^** \| **Sensitivity^a^** \| **Specificity^a^** \| **AUC(95% CI)** \| **Z** \| **P**; Day1(n=92) \| \| \| \| \| \| \|; Model 1 \| 0.783 \| 84.78% \| 83.56% \| 89.47% \| 0.917(0.854,0.980) \| 13.058 \| <0.001; Model 2 \| 0.930 \| 85.87% \| 82.19% \| 100.00% \| 0.963(0.928,0.997) \| 26.623 \| <0.001; Day2-Day5(n=104) \| \| \| \| \| \| \|; Model 1 \| 0.818 \| 65.39% \| 59.76% \| 86.36% \| 0.753(0.644,0.863) \| 4.538 \| <0.001; Model 2 \| 0.782 \| 79.81% \| 76.83% \| 90.91% \| 0.904(0.844,0.963) \| 13.228 \| <0.001; >Day5(n=85) \| \| \| \| \| \| \|; Model 1 \| 0.704 \| 71.77% \| 74.19% \| 65.22% \| 0.687(0.553,0.821) \| 2.738 \| 0.006; Model 2 \| 0.731 \| 82.35% \| 79.03% \| 91.30% \| 0.906(0.843,0.969) \| 12.624 \| <0.001; Model 1 included initial rhythm, APACHE II score, pupillary reflex, and NSE. \| \| \| \| \| \| \|; Model 2 included the SERS based on Model 1. ^a^ Results from leave-one-out cross-validation. \| \| \| \| \| \| \| | | | | | | | | | | | | | | |
| **Supplemental Table 9 Comparison of Predictive Performance for Poor Neurological Outcome Among Different Models After Excluding Patients Who Underwent WLST** | | | | | | | | | | | | | | |
| **Indicator** | **AUC Difference (95%CI)^a^** | | **P** | | **IDI(95%CI)^a^** | | **P** | | **NRI(95%CI)^a^** | | **P** | |  | |
| Day1(n=92) |  | |  | |  | |  | |  | |  | |  | |
| Model 2 vs. Model 1 | 0.045(0.000,0.090) | | 0.048 | | 0.331(0.247,0.415) | | <0.001 | | 1.516(1.198,1.833) | | <0.001 | |  | |
| Day2-Day5(n=104) |  | |  | |  | |  | |  | |  | |  | |
| Model 2 vs. Model 1 | 0.153(0.055,0.251) | | 0.002 | | 0.201(0.104,0.299) | | <0.001 | | 0.984(0.584,1.385) | | <0.001 | |  | |
| >Day5(n=85) |  | |  | |  | |  | |  | |  | |  | |
| Model 2 vs. Model 1 | 0.220(0.097,0.344) | | <0.001 | | 0.305(0.190,0.421) | | <0.001 | | 1.123(0.717,1.530) | | <0.001 | |  | |
| Model 1 included initial rhythm, APACHE II score, pupillary reflex, NSE. Model 2 included the SERS based on Model 1. | | | | | | | | | | | | | |  |
